# Prognostic nomogram for hepatocellular carcinoma in adolescent and young adult patients after hepatectomy

**DOI:** 10.18632/oncotarget.18192

**Published:** 2017-05-23

**Authors:** Wei Zhang, Yifei Tan, Shu Shen, Li Jiang, Lunan Yan, Jiayin Yang, Bo Li, Tianfu Wen, Yong Zeng, Wen Tao Wang, Mingqing Xu

**Affiliations:** ^1^ Department of Liver Surgery, Liver Transplantation Center, West China Hospital of Sichuan University, Chengdu, Sichuan Province, China

**Keywords:** adolescent and young adult oncology, hepatocellular carcinoma, nomogram, liver resection

## Abstract

**Background:**

Hepatocellular carcinoma (HCC) was rarely discussed in adolescent and young adult (AYA) patients. This study aimed to discuss the character of AYA HCC patients and establish an effective prognostic nomogram for patients after hepatectomy.

**Results:**

For all of the patients, the median OS was 57 months with 5-year OS rate 60.4%, and DFS was 48 months with 5-year DFS rate 51.4%. The tumor size, vascular invasion status and the pathological differentiation were the independent predictors for both OS and DFS. Except for that, gender, Neutrophil-lymphocyte ratio, HbeAg, and α-Fetoprotein were the predictors for OS. The c-index for OS prognostic nomogram was 0.75 (95% CI, 0.71 to 0.79), and c-index was 0.70 (95% CI, 0.66 to 0.74) for DFS prognostic nomogram, which was better than American Joint Commission on Cancer 2002 and 2010, Okuda staging system, the Japanese Integrated Staging system, and Tokyo staging system.

**Materials And Methods:**

This study was based on 423 AYA HCC patients (younger than 40 years old) undergoing hepatectomy in West China Hospital between 2008 to 2014. Based on the multivariate risk factors, the nomogram was constructed for predict the possibility for overall survival (OS) and disease-free survival (DFS) rate. Harrel’s concordance index (c-index) was used to compare the predictive accuracy and discriminative ability between the nomogram and eight contemporary staging systems.

**Conclusions:**

Our prognostic nomogram could accurately and preciously provide individual prediction for AYA HCC patients in OS and DFS after hepatectomy.

## INTRODUCTION

Hepatocellular carcinoma (HCC) is the most common primary liver malignancy, which remains increasing in recent years [1]. The adolescent and young adult (AYA) oncology patients group, defied as individuals younger than 40 years old according to the National Comprehensive Cancer Network (NCCN), is a special group that has not shared the same survival improvement seen in the last 30 years comparing to the older and younger patients which was shown by the AYA Oncology Progress Review Group in 2006 in United States [2]. Although occupying less in the whole population, AYA HCC patients were believed with the characters of higher malignant degree, later diagnosis and poorer outcome. However, it has barely studied previously, especially for the patients younger than 40 years old, which sparkles our inspiration to undertake this study.

Because of the shortage of the liver donor for liver transplantation, hepatectomy was still the most common curative approach to treat HCC [3, 4]. But the surgical outcome was still relied on the different stage of tumors [5]. For those reasons, several studies are trying to divide the HCC patients into different stages reflecting the contrast in outcomes of patients in recent years [6–12]. Nomogram have been accepted as reliable tools to integrate important risk factors and predict the outcome for oncology prognosis [13–16]. And at the same time, the accuracy could be texted by concordance index and calibration curve comparing to other staging systems [17]. More importantly, the graph could provide prognostic information both for groups or individual, which means that it could be used for both doctors and patients to calculate the survival rate. In our study, we aim to evaluate the characteristics of AYA HCC patients and try to create a new staging system of nomogram to predict the outcome of the special group.

## RESULTS

### Clinicopathologic characteristics

Four hundred and twenty-three AYA patients meeting the inclusion were involved in this study. The characteristics of all the patients were listed in Table 1 and Table 2. Nearly 79.9% of the patients were male and the mean age was 36 years. For all of those patients, 378 AYA patients have a history of hepatitis B virus infection which remains HbsAg positive and among them, 95 patients were HbeAg positive and 152 patients had HBV-DNA level of more than 10^4^ copies/ml. Although 74.2% of the patients were diagnosed as liver cirrhosis, majority of patients have an acceptable liver function with child-pugh A status (*n* = 400). The cut-off of the neutrophil-lymphocyte ratio (NLR) was 2.8. For 334 patients, α-Fetoprotein (AFP) was observed with an increase more than 20 ng/ml, and of which, AFP increased significantly in 277 patients (≥ 200 ng/ml).

**Table 1:** Participant characteristics of categorical variables

Categorical variables	*N* (%)	Median survival (months)	Chi values	*p*
**Gender**			2.951	0.086
** Male**	338 (79.9)	55.7		
** Female**	85 (20.1)	61.4		
**Liver cirrhosis**			1.714	0.190
** Negative**	109 (25.8)	61.5		
** Positive**	314 (74.2)	55.3		
**Platelets**			3.326	0.068
** <100**	76 (18.0)	49.7		
** >100**	347 (82.0)	55.1		
**Neutrophil-lymphocyte ratio (NLR)**			30.910	< 0.001
** < 2.8**	251 (59.3)	64.2		
** ≥ 2.8**	172 (40.7)	35.2		
**HbsAg**			4.045	0.044
** Negative**	45 (10.6)	67.7		
** Positive**	378 (89.4)	55.5		
**HbeAg**			6.981	0.008
** Negative**	328 (77.5)	59.4		
** Positive**	95 (22.5)	38.5		
**HBV DNA, copies/ml**			3.719	0.054
** < 104**	271 (64.1)	60.0		
** ≥ 104**	152 (35.9)	50.0		
**α-Fetoprotein (AFP), ng/ml**			20.229	< 0.001
** < 200**	146 (34.5)	68.6		
** ≥ 200**	277 (65.5)	50.0		
**Tumor Number**			5.532	0.019
** Single**	305 (72.1)	60.0		
** Multiple**	118 (27.9)	44.0		
**Vascular invasion**			61.579	< 0.001
** Negative**	263 (62.2)	67.7		
** Micro**	71 (16.8)	39.7		
** Macro**	89 (21.0)	29.7		
**Lymphnode**			2.403	0.121
** Negative**	358 (84.7)	56.9		
** Positive**	65 (15.3)	54.1		
**Edmondson-Steiner classification**			24.514	< 0.001
** I and II**	219 (51.8)	65.5		
** III and IV**	204 (48.2)	46.6		

**Table 2:** Participant characteristics of continuous variables and its connection with survival rate

Continuous variables	Median	IQR	OR	CI	*p*
**Age, year**	36.03	31.98–38.29	1.004	0.969–1.040	0.817
**BMI**	22.35	20.20–23.99	0.951	0.896–1.010	0.099
**Hemoglobin, g/L**	144	130–154	0.997	0.989–1.006	0.526
**Total bilirubin, umoll/L**	13.8	10.3–19.3	1.002	0.997–1.008	0.397
**AST, U/L**	43	31–71	1.000	0.999–1.001	0.518
**ALT, U/L**	45	31–69	1.001	0.999–1.002	0.540
**Creatinine, mmoll/L**	74.2	63.6–83.6	0.994	0.983–1.006	0.328
**PT, seconds**	11.8	11.1–12.6	1.048	0.932–1.178	0.435
**INR**	1.1	1–1.1	1.974	0.580–6.715	0.276
**Tumor Size, cm**	6	3.5–10	1.153	1.111–1.196	0.000

Abbreviations: IQR = interquartile range; OR = odds ratio; CI = confidence interval; BMI = body mass index; AST = aspartic aminotransferase; ALT = alanine aminotransferase; PT = prothrombin time; INR = international normalized ratio

The median tumor size was 6 cm, and 72.1% of the tumor occurred as the single form. Majority of patients (62.2%) did not have the evidence of the MIVI and MAVI, and the poorer and the better differentiation histologic grade based on the Edmondson-Steiner (ES) classification occupied equally in those patients (51.8% vs 48.2%).

### Survival outcomes

**Figure 1:**
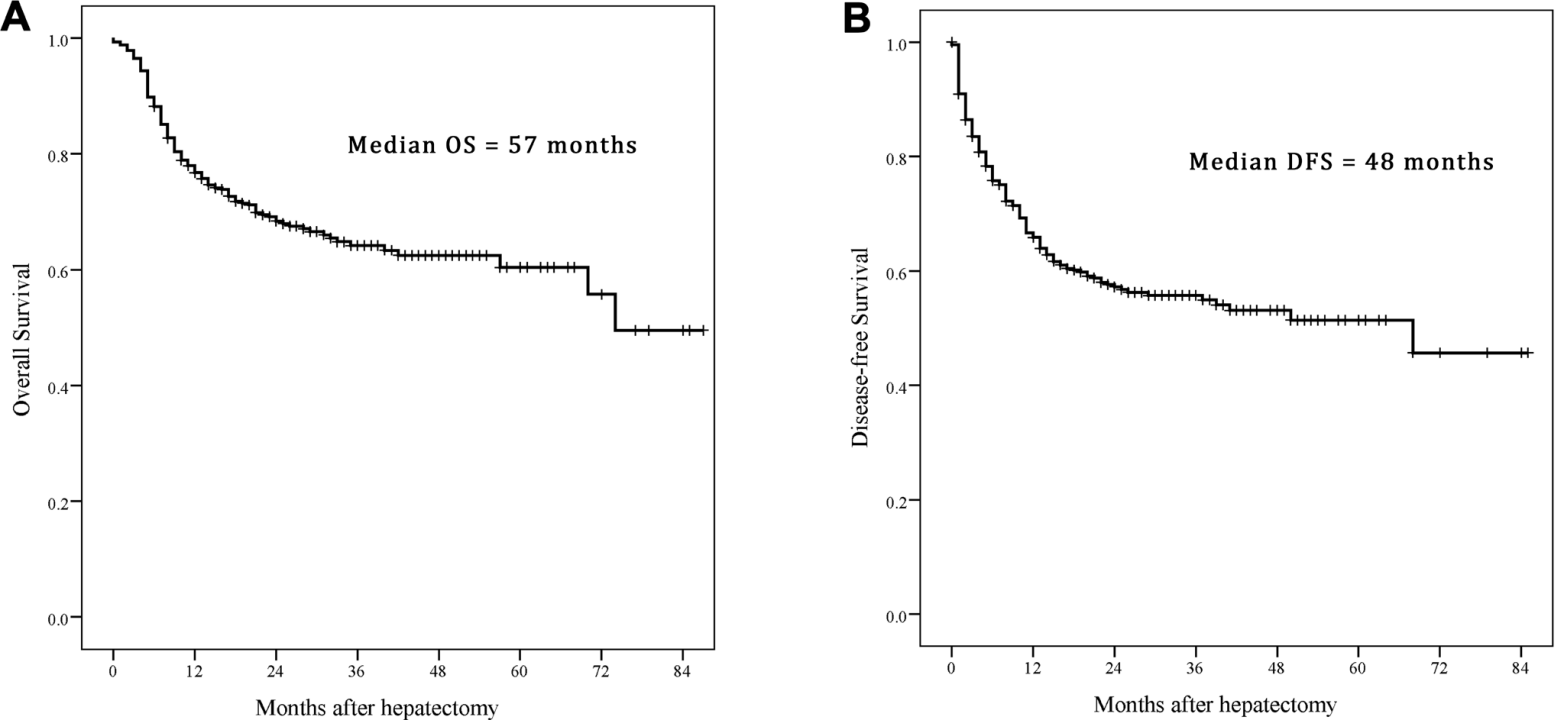
Kaplan–Meier estimate the survival rate of adolescent and young adult (AYA) hepatocellular carcinoma (HCC) patients (**A**) Overall survival (OS); (**B**) disease-free survival (DFS).

For all of the patients, the median OS was 57 months with 5-year OS rate 60.4%, and DFS was 48 months with 5-year DFS rate 51.4% (Figure 1A, 1B). The results of the univariate analysis were listed in Table 1, lower NLR (< 2.8 vs ≥ 2.8, *p* < 0.001), negative HbeAg (vs positive, *p* = 0.008), lower AFP (< 200 vs ≥ 200, *p* < 0.001), negative vascular invasion (*p* < 0.001), less tumor number (single vs multiple, *p* = 0.019) and better differentiation tumor grade (I/II vs III/IV, *p* = 0.034) were associated with a better prognosis of OS. Moreover, the tumor size was also an important influence to the survival as shown in Table 2 (*p* < 0.001). All significant factors in univariate analysis and other clinical meaningful data like gender, HBV DNA and platelets were entered into the multivariate analysis which was expressed as hazard ratio (HR) and 95% confidence interval (CI) as shown in Table 3 for OS and Table 4 for DFS. Finally, the male (1.661 [1.05–2.64]), higher NLR (1.61 [1.10–2.36]), positive HbeAg (1.47 [1.01–2.13]), higher AFP (1.64 [1.08–2.51]), larger tumor size (1.10 [1.05–1.14]), vascular invasion (MIVI vs negative, 1.76 [1.12–2.76], MAVI vs negative, 2.27 [1.50– 3.41]), and poorer ES differentiation (1.48 [1.03– 2.13]) were associated with the worse OS. For DFS of the patients, the larger tumor size (1.07 [1.03–1.12]), vascular invasion (MIVI vs negative, 1.54 [0.99–2.38], MAVI vs negative, 3.77 [2.67–5.44]), and poorer ES differentiation (1.45 [1.06–1.98]) were the main impacts.

**Table 3:** Multivariate logistic regression analysis of overall survival rates

Variable	BETA	*p*	Hazard ratio(HR)	95% confidence interval of HR
Gender, male vs female	0.508	0.032	1.66	1.05–2.64
NLR, ≥ 2.8 vs < 2.8	0.477	0.014	1.61	1.10–2.36
HbeAg, positive vs negative	0.382	0.045	1.47	1.01–2.13
AFP, g/L, ≥ 200 vs < 200	0.496	0.022	1.64	1.08–2.51
Size	0.091	0.000	1.10	1.05–1.14
Vascular invasion				
Microvascular invasion vs Negative	0.563	0.015	1.76	1.12–2.76
Macrovascular invasion vs Negative	0.817	0.000	2.27	1.50–3.41
Edmondson-Steiner classification, III/IV vs I/II	0.392	0.034	1.48	1.03–2.13

**Table 4:** Multivariate logistic regression analysis of disease-free survival rates

Variable	BETA	*p*	Hazard ratio (HR)	95% confidence interval of HR
Size	0.071	0.000	1.07	1.03–1.12
Vascular invasion				
Microvascular invasion vs Negative	0.428	0.056	1.54	0.99–2.38
Macrovascular invasion vs Negative	1.327	0.000	3.77	2.67–5.44
Edmondson-steiner classification, I/II vs III/IV	0.371	0.019	1.45	1.06–1.98

### Survival nomogram

**Figure 2:**
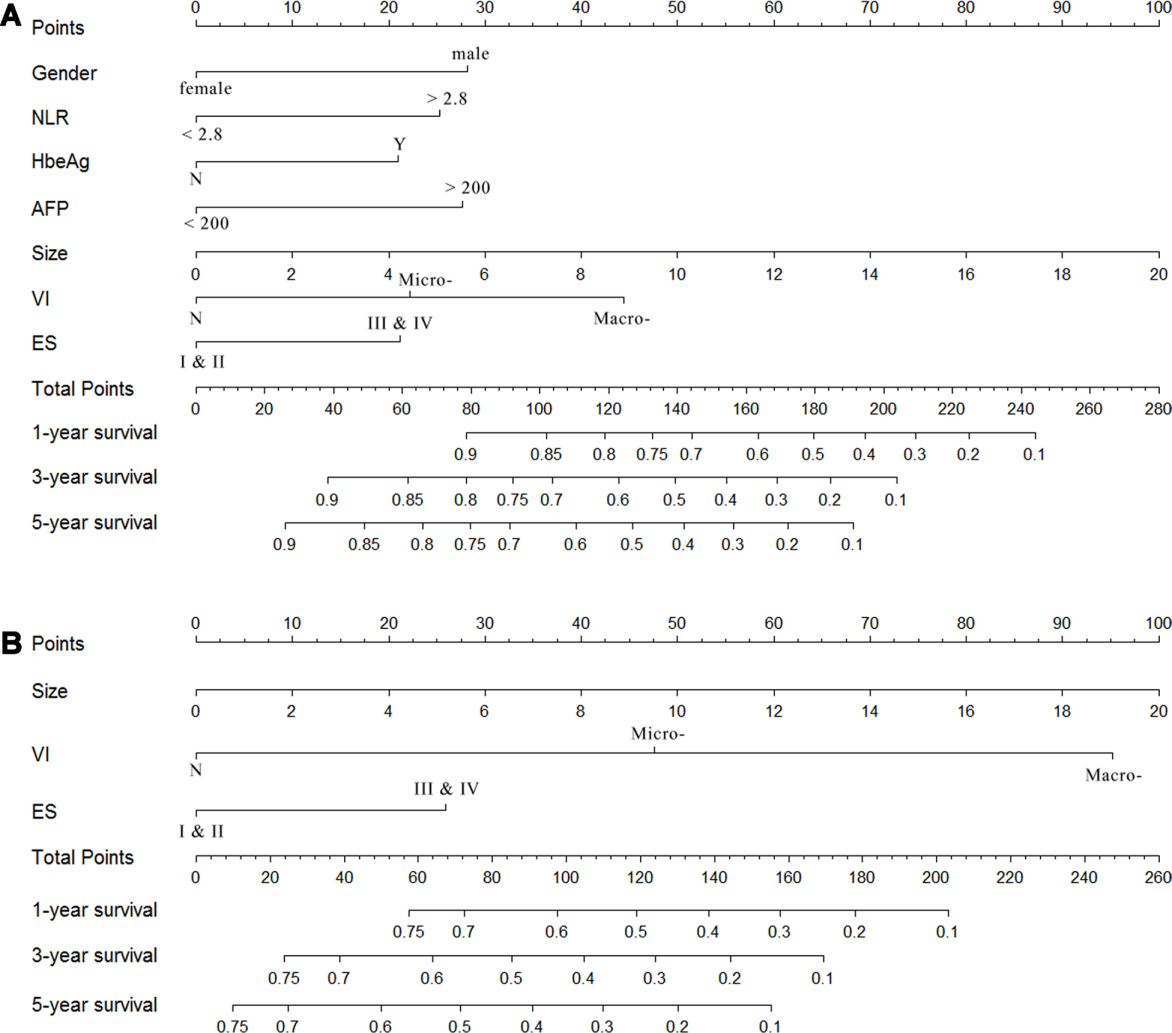
Survival nomogram of adolescent and young adult (AYA) hepatocellular carcinoma (HCC) patients (**A**) Overall survival (OS) nomogram; (**B**) disease-free survival (DFS) nomogram. NLR, Neutrophil-lymphocyte ratio; AFP, α-Fetoprotein; VI, vascular invasion, including microvascular invasion and macrovascular invasion; ES, Edmondson-Steiner classification. (For clinical use of the model, based on the points that each variable achieved from individual patients, the total scores would be calculated according to the nomogram, and the probability of survival rate could be determined.)

**Figure 3:**
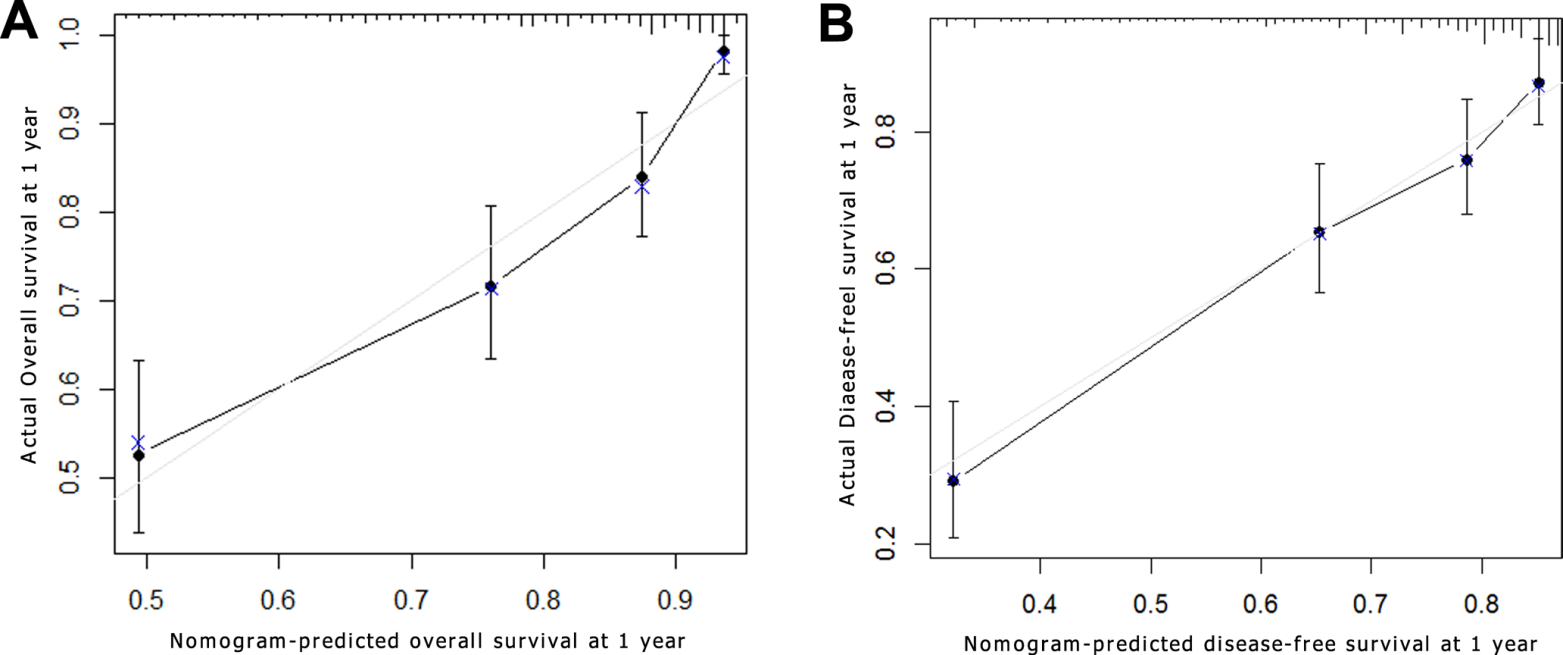
Survival nomogram calibration curve of adolescent and young adult (AYA) hepatocellular carcinoma (HCC) patients Nomogram-predicted survival at 1 year is plotted on X axis; actual survival at 1 year is plotted on the y axis. (**A**) Overall survival (OS); (**B**) disease-free survival (DFS).

Based on the results of the multivariate analysis, the OS and DFS prognostic nomograms were built respectively after integrating all the significant independent factors in Figure 2A and 2B. The c-index for OS prognostic nomogram was 0.75 (95% CI, 0.71 to 0.79), and c-index was 0.70 (95% CI, 0.66 to 0.74) for DFS prognostic nomogram. A calibration curve plotting was drawn based on the nomogram-predicted OS and DFS at 1 year on the X-axis and the rates of OS and DFS calculated by the Kaplan–Meier method at 1 year on the Y-axis (Figure 3A and 3B). The calibration plots for 1-year survival for both OS and DFS were well matched the ideal 45-degree line, indicating acceptable calibration.

### Comparison between the nomogram and conventional staging system

**Figure 4:**
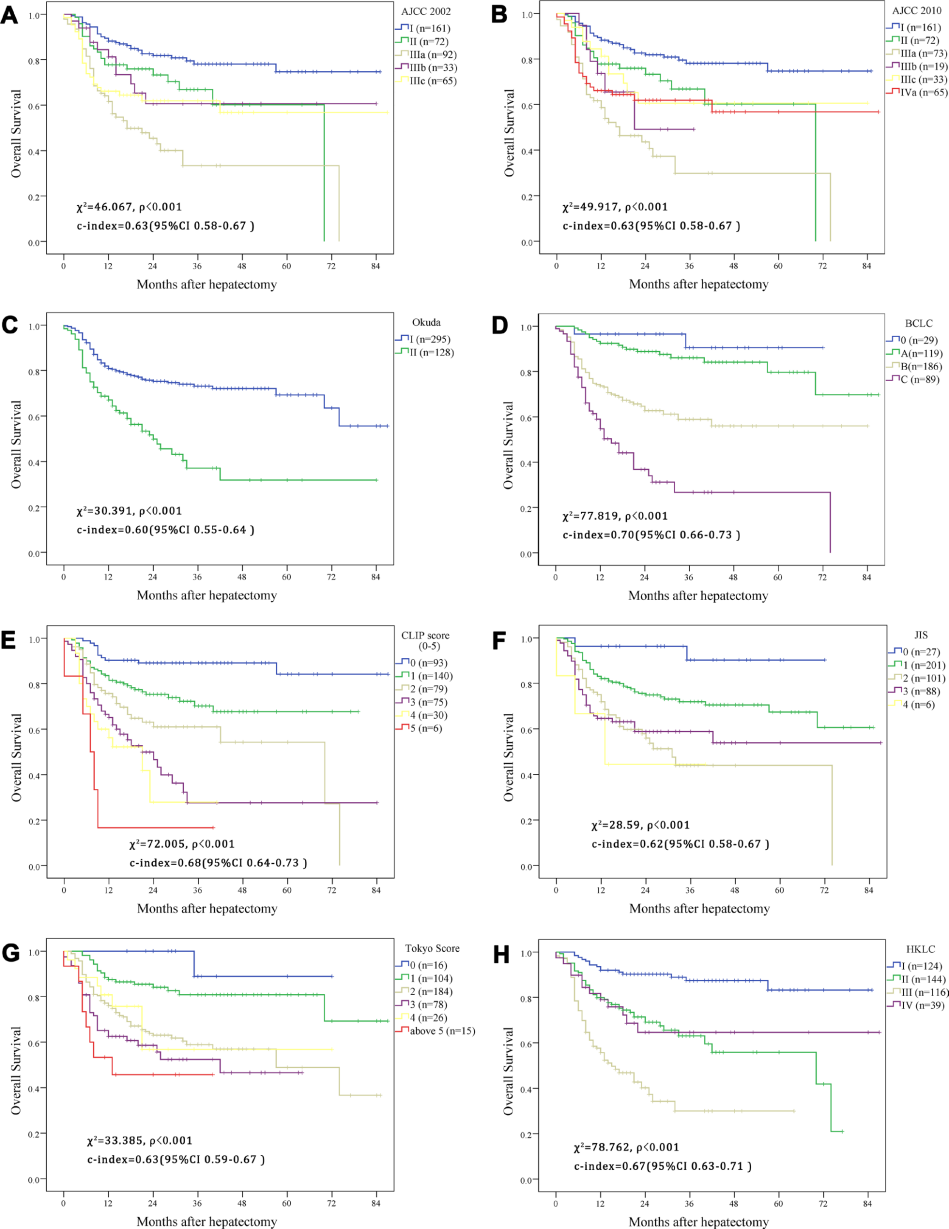
Kaplan–Meier estimate the survival rate of adolescent and young adult (AYA) hepatocellular carcinoma (HCC) patients based on different contemporary staging systems (**A**) American Joint Commission on Cancer (AJCC) 2002 edition; (**B**) AJCC 2010 edition; (**C**) the Okuda staging system; (**D**) the Barcelona Clinic Liver Cancer staging system; (**E**) the Cancer of the Liver Italian Program staging system; (**F**) the Japanese Integrated Staging system; (**G**) the Tokyo Score system; (**H**) the Hong Kong Liver Cancer classification system.

**Figure 5:**
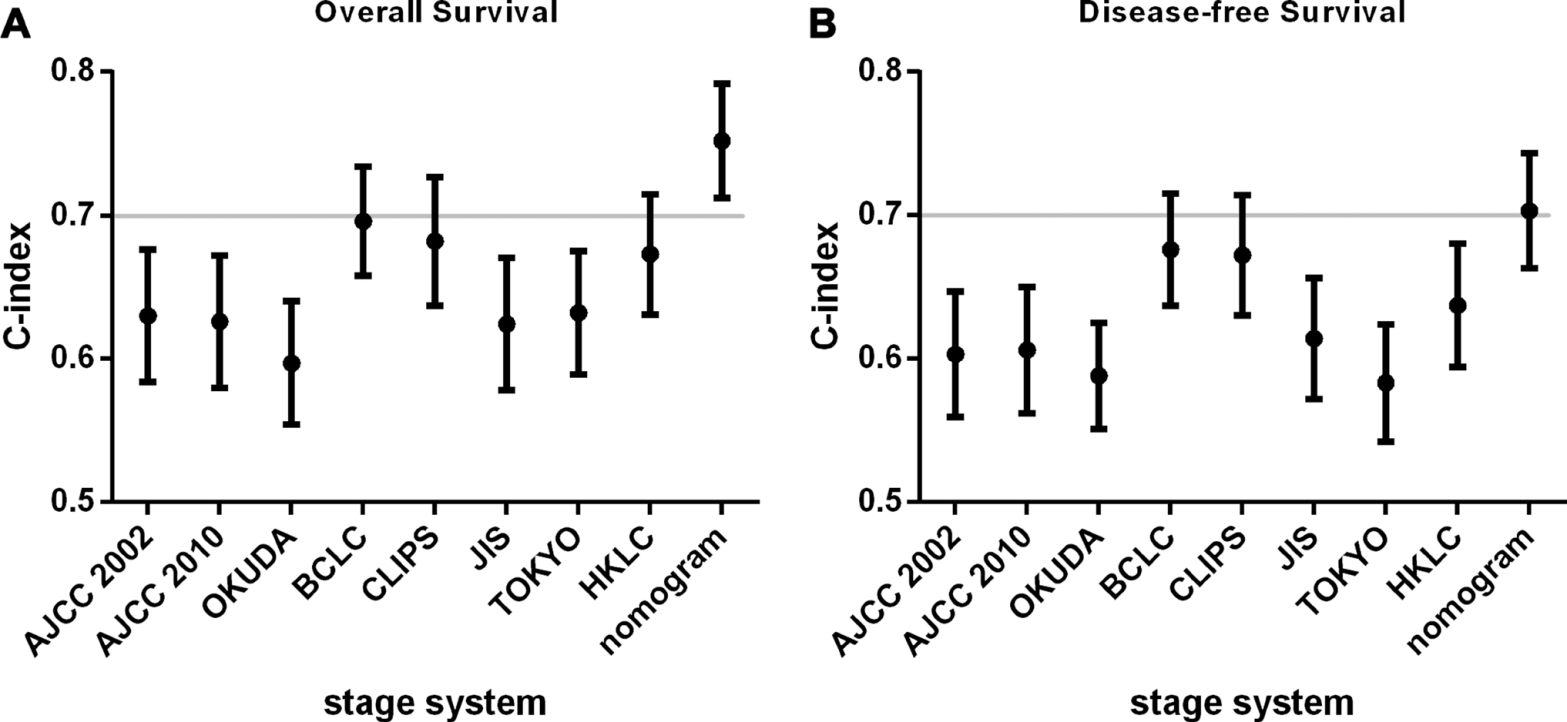
Concordance index in different staging systems (**A**) Overall survival (OS); (**B**) disease-free survival (DFS). AJCC, American Joint Commission on Cancer; BCLC, the Barcelona Clinic Liver Cancer staging system; CLIPS, the Cancer of the Liver Italian Program staging system; JIS, the Japanese Integrated Staging system; HKLC, the Hong Kong Liver Cancer classification system.

As shown in Figure 4, all the staging system have a good prognostic stratification for all of the AYA patients (*p* < 0.001). Despite for the acceptable OS results that the classification provided, overlap could be found in some stage in the survival plotting, especially the intermediate stage such as I and II in AJCC and HKLC stage system, which meant that the distinguish between those mediate stages could not predict the prognostic outcome perfectly. The c-index were calculated respectively according to the different stage system, and compared with the nomogram for OS and DFS in the Figure 5A and 5B. The nomograms both in OS and DFS had a better prognostic effect, which was the only model whose c-index was more than 0.7. However, comparing to the BCLC, CLIP and HKLC stage, it had no significant differences to the nomograms even with a higher c-index (*p* = 0.1, 0.1, 0.06 in OS and *p* = 0.48, 0.45, 0.11 in DFS in BCLC, CLIP and HKLC, respectively). While for the rest five stage system (AJCC 2002, AJCC 2010, OKUDA, JIS, Tokyo stage system), the c-index of the nomogram was found to be significantly better (*p* < 0.01 in OS and *p* < 0.05 in DFS for all of the five comparisons).

## DISCUSSION

Due to the shortage samples diagnosed as HCC in many oncological institutes, few studies had focused on the survival condition and risk factors for the younger patients, particularly. Thereafter, we designed the retrospective study for the AYA HCC patients younger than 40-year old, trying to find out more factors affecting the survival and creating a new prognostic model for these special group.

As far as we concerned, this is one of the largest cohort concentrating on the AYA HCC patients. Comparing to the younger and elder patients, AYAs are different in their biological, psychosocial and socio-behavioral characteristics and thereafter represented a unique oncological population [18]. In some published studies, the AYA HCC patients tended to have a poor prognosis because of advanced stage [19]. While for majority of the research, the AYA HCC patients shared the similar OS and DFS with the elder patients and age itself was not an independent prognostic factor if the stages were matched [20, 21]. However, the samples involved in those study were usually scarce which might result in the statistical bias. But one thing for sure is that most AYAs patients had a history of hepatitis infection and a good liver function which resulted in the detection of tumors lately, but instead, increasing the resection rate while decreasing the complication rate [19–23]. In our present study, 89.4% of the patients had a positive HbsAg immunology and 94.6% had a liver function with child-pugh A. However, majority of the patients (65.4%) were diagnosed as advanced stage of BCLC B and C with 5-year survival rate 56.0% and 26.7%, respectively. While for those diagnosing as early stage, the 5-year survival rate could achieve to 90.5% in stage 0 and 79.7% in stage A, which was similar to the previous study [8]. These results suggest that if the diagnosis was detected earlier, the AYA HCC patients could have a favorable outcome. And periodic surveillance especially for those chronic hepatitis B carriers was an important part for the early detection.

To quantify different risk factors influencing the outcome of the HCC patients, several institutes and organizations tried to propose staging system and stratify patients into different degrees which could representative the outcome of their characters. In our present study, we listed eight commonest stage system and compared the accuracy and effect with our new nomogram. But it must be stated that not all of the classifications were originally developed as the prognostic model. For some, like Okuda, BCLC, HKLC, CLIP and JIS, were trying to help clinics to confirm the treatment approach, while for another, involving the operative and pathological factors, like AJCC and our prognostic nomogram, are used to ascertain the long-term survival after surgery. Regardless, we applied all the stage systems to predict the outcome of OS and DFS of HCC patients after hepatectomy, and the survival was classified based on different stage and analyzed by the Kaplan–Meier method. Moreover, the Harrell’s concordance index was used to measure the prognostic capacity of different stage and to assess how well the model performed. Our nomogram indicated a better predictive ability with c-index of 0.74 and 0.70 comparing to five stage systems both in OS and DFS, and even with no statistical difference, the c-index of the nomogram was still higher than it of the BCLC, CLIP and HKLC. Besides, majority of contemporary standard model existing some shortages with a great deal of heterogeneity within each risk factors. Nomogram was a new method not only reflect the predictive value for each variable but also the complex interaction with the other variables [16]. Moreover, nomogram are the visualizations of the quantized risk variables which was available not only for the surgeons but for each individual patient to understand the short- and long-term outcome.

In our present study, the tumor size, vascular invasion and pathological differentiation were recognized as the variables both in nomograms for OS and DFS, which were demonstrated associating with outcomes by previous studies [24–26]. And among those, tumor size was regarded as the most important factor influencing the outcome of patients, referring to all of those contemporary eight staging systems. MAVI of the tumor defining as the invasion to the main branch of the vessel or the thrombosis in the vessels, was thought to be unresectable in some staging system, and TACE was recommended as the best choice for those patients [8]. However, the benefit of liver resection was proved with a better survival rate comparing to TACE in several studies [27, 28]. If the portal vein thrombosis was stratified according to the invasion of the portal branch, hepatectomy provided survival benefit for patients especially for the tumors limited in the lateral branch of the portal vein not exceeding to the main portal vein and the superior mesenteric vein [27]. More recently, Kokudo T et al. [28] undertook a multicenter, nationwide study of 6474 HCC patients with portal vein tumor thrombosis as well and demonstrated that hepatectomy was associated with a longer survival outcome than non-surgical treatment if the thrombosis was limited to the first order branch. MIVI was associating with the aggressive behavior of HCC and demonstrating with a worse survival outcome nowadays [24]. Many efforts have been made on preoperative assessment of microvascular invasion over past decades, expecting to add it to the new inclusion for treatment of HCCs [13]. In our study, we stratified the vascular invasion into MAVI and MIVI, which would be more suitable and accurate for decision and judgement for clinical use.

Gender was considered as one of the variables influencing the long-term OS in the present study, but which was without statistical difference in univariate analysis (*p* = 0.068). This would be related to the high percentage of male patients in this study (79.9%) and associated with the high prevalence rate of male HCC patients in the whole population [1]. The presence of HbeAg in serum indicates active viral replication in hepatocytes and also was considered as an indicator for antiviral-drug treatment combined with HBV DNA [29]. Moreover, some studies showed that positive HbeAg may promote intrahepatic metastasis by changing tumor microenvironment and resulting in early recurrence after liver resection [30]. AFP was long demonstrated increasing in the occurrence of the HCCs and had a great value in diagnosing HCCs. Except that, PIVKA-II and AFP-L3 was also proved to be the tumor markers increasing the accuracy of the diagnosis for HCCs especially for the AFP-negative patients [31]. Moreover, previous study had demonstrated preoperative AFP was related to the poor prognosis for HCCs, and involved into the CLIP staging systems [9]. In our study, we regarded AFP 200 ng/dL to stratify the patients, and in the univariate analyses, the lower group occupied a longer survival rate comparing to the higher group (*p* < 0001). But the impact of AFP was still controversial, some studies also showed there was no prognostic value for the tumor less than 3 cm [32, 33]. Future studies are still needed to identify the mechanisms association between preoperative AFP levels and HCC progression. NLR, a marker of systemic inflammation, was considered a prognostic factor in predicting the outcome of HCC patients after liver transplantation and liver resection [34–36]. High NLR was associated with a high infiltration of tumor-associated macrophages, which promote systemic neutrophilia, and thereafter associating with aggressive phenotype of HCC, favoring tumor vascular invasion and suppressing the host immune surveillance [36]. More recently, Yang et al. [35] conducted a study of 526 HCC patients undergoing liver resection and proposed that preoperative NLR ≥ 2.81 may be an indication of poor DFS and OS. Similarly, in our cohort, higher NLR patients (NLR ≥ 2.8) have a shorter survival time comparing to the lower group (NLR < 2.8) with 35.2 months and 64.2 months, respectively (*p* < 0.001), and therefore was considered as a main risk factor in the OS nomogram that we proposed. Tumor number was considered as one of the most important factors affecting the long-term survival of the HCC patients and was involved in most of the contemporary staging systems [6, 8, 10–12]. We considered the multiple occurrence HCCs associating with a poor survival rate comparing to the single tumor in the univariate analyses (*p* = 0.019). While the tumor number may not be a prognostic factor in the multivariate analyses for its relationship to the aggression of tumors which may connect with vascular invasion and pathological differentiation.

To the best of our knowledge, this is the first nomogram focusing on the AYA patients based on a large database with long-term follow-up. However, there are some limitations in our study. Firstly, it is a retrospective, single center study, which might case some bias when selecting the patients undertaking hepatectomy. In this cohort, majority patients have a history of hepatitis B infection, while for other countries, the prevalence was different, and even minority of patients have a status of liver cirrhosis due to hepatitis infection. Because the number of patients diagnosing as hepatitis C was scarce, especially for the AYA cohort, the nomogram may not be such applicable for the hepatitis C infection patients. Secondly, due to the lower prevalence of AYA patients, we did not design a validation group to verify the result of our nomogram. At last, because of the different geographic and institutional heterogeneity existing among patients with HCCs, it will certainly be necessary to validate this prognostic nomogram at other institutions. We hope several large-samples oncological centers would contribute to validate the nomogram, making the nomogram be more useful for future clinical trial stratification or assessment of AYA patients treatment and prognosis.

In conclusion, we established two prognosis nomograms to predict the OS and DFS in AYA HCC patients based on the largest young HCC patient cohort. And in this nomogram, the tumor stage like tumor size, vascular invasion and pathological differentiation, the tumor marker, like AFP and NLR, the gender and etiology of AYA patients were associated with the long-term survival. Through this model, clinicians could estimate the post-operative survival of individual AYA patients more preciously and thereafter provide the guidance for the frequency of post-operative surveillance as well as adjuvant therapy in patients with high risk of recurrence.

## MATERIALS AND METHODS

This study was approved by the West China Hospital Ethics Committee, and in accordance with the ethical guidelines of the Declaration of Helsinki.

### Study design

Between December 2008 and December 2014, four hundred and sixty AYA patients underwent hepatectomy in West China Hospital, Sichuan University, Chengdu, China. The data of all patients were retrospectively reviewed. Inclusion criteria included the age of patients (16–40 years old), no history of previous anticancer therapy, radical resection of macroscopic liver tumors and the histopathologically proven HCC. Exclusion criteria included the mixed type of liver cancer, simultaneously underwent resection and radiofrequency ablation and palliative resection. Finally, 423 AYA patients were involved in this study.

### Preoperative examination and indications for hepatectomy

Laboratory blood examination including routine blood test, liver and renal function, coagulation tests, hepatitis B and C immunology and tumor markers were completely tested. Hepatitis B virus (HBV) infection was identified if serum hepatitis B surface antigen (HbsAg) positively, and moreover the HBV DNA load was examined to judge whether the antiviral drug should be taken and determine the long-term of the effect of the antiviral drug. Preoperative chest X-ray, electrocardiogram (ECG), ultrasonography and enhanced CT or MRI of abdomen to exclude the cardiopulmonary diseases and confirm the HCC diagnosis and surgery approach. Preoperative diagnosis was judged by 2 types of clinical imaging in addition to a high serum level of α-fetoprotein (AFP) and a background of hepatitis infection. All the images were evaluated by two experienced radiologists independently and the discussion would be detected if any controversies were existed to generate the final report. Besides, appropriate residual liver volume (RLV) need to be assessed by surgeons to avoid the postoperative liver failure. Generally speaking, for patients without liver cirrhosis, 30% RLV after hepatectomy was adequate and more than 50% should be guaranteed if the liver cirrhosis was combined. Thereafter our institute recommend the indications for hepatectomy were as follows: lack of ascites, gastric esophageal varices bleeding, presence of Child-Pugh A of B liver function, and appropriate RLV [37]. Since ICG-15 was not routinely detected for all the HCC patients, the ICG-15 criteria was not taken as the indicators for hepatectomy.

Postoperative HCC diagnosis was confirmed by histopathological examination for all the patients. Two experienced pathologists confirmed the final diagnosis and all surgical specimens were routinely examined for the presence of the microvascular invasion. Curative hepatic resection was defined if grossly complete removal of all detected tumors and tumor-free margins confirmed by histopathology [38, 39]. Microvascular invasion (MIVI) was defined as the presence of tumor only was visible on microscopy in a portal vein, hepatic vein, or a large capsular vessel of the surrounding hepatic tissue lined by endothelium [13, 40], and the macrovascular invasion (MAVI) was described as the thrombus was found in the main branch of the vessel in preoperative image or intra-operation. All the specimens were staging by Edmondson-Steiner grading after hepatectomy [41].

### Follow-up and postoperative treatment

Liver and renal function test, routine blood test, serum AFP assay and ultrasonography were performed once in the first month after resection and then checked every 3 months in the first postoperative year and every 6 months in the subsequent years. If the recurrence was suspected, the enhanced CT or MRI was performed. Tracing and follow-up of all the patients was until June 1st 2016. Overall survival (OS) and disease-free survival (DFS) were the endpoints of our study. OS was calculated from the date of hepatectomy to the date of patient’s death or the date of last follow-up visit. DFS was measured from the date of hepatectomy to the date when tumor recurrence was diagnosed.

If the recurrence of tumor was confirmed, the treatment for HCC was similar to the initial HCC. For the tumors were found in intrahepatic areas, hepatectomy was considered if the patients can tolerate the surgery based on the liver function and residual liver volume according to the indications for hepatectomy as used at the time of curative resection [42]. RFA was also a kind of common therapy if the patients refused the second laparotomic surgery or the lesions recurred were smaller than 3 cm which was advocated in BCLC A stage patients [26]. If the curative approach could not be performed because of poor liver function or other unexpected factors, the transhepatic arterial chemotherapy and embolization (TACE) or sorafenib therapy were applied.

### Categorization of patients in different staging system

Patients were categorized according to the eight contemporary HCC staging systems including the sixth and seventh editions of American Joint Commission on Cancer (AJCC 2002 and 2010) [6], the Okuda staging system [7], the Barcelona Clinic Liver Cancer (BCLC) staging system [8], the Cancer of the Liver Italian Program (CLIP) staging system [9], the Japanese Integrated Staging (JIS) system [10], the Tokyo Score system [11], and the Hong Kong Liver Cancer (HKLC) classification system [12]. Due to the lack of samples of some groups, we combined the score 5, 6, and 7 in Tokyo Score into score above 5 group, and the I a and II b stage in HKLC group were generally described as II stage, and the similar method was used in III and IV stage in HKLC group.

### Statistical analysis

Categorical variables were expressed as count and calculate the median survival respectively, and continuous variables were described as median and interquartile range. According to the eight major existing HCC staging system, relative clinical variables were used to stage patients. The cut-off of some continuous data, like NLR, was determined by receiver operating characteristic curve (ROC) method, in which the best cut-off could be achieved with the best sensitive and specific rate. Survival outcomes were determined by Kaplan–Meier methodology, and compared with the log-rank test. Univariate and multivariate analyses were performed using the Cox proportional hazard model to acquire the prognostic factors for survival. A nomogram was formulated by R, version 3.3 (https://www.r-project.org/) according to the result of the multivariate analyses, and the methods was referred to the studies published previously [14, 15]. A calibration curve was generated to describe the agreement between nomogram-predicted probability of both overall and disease-free survival at 1 years. The ability of each staging system to stratify post-operative survival was quantified using Harrell’s concordance index (c-index) which was published previously [16, 43], and the calibration was generated by 1000 bootstrap samples to decrease the bias. The larger the c-index was, the more accurate the prognostic prediction was achieved. And moreover, if the c-index is more than 0.7, the model could be more accurate comparing to the lower one. A *p* value < 0.05 was considered to be statistical significance for all the analyses. The statistical analyses of the collected data were performed with the SPSS 19.0 statistical software (IBM, USA) and R, version 3.3.

For clinical use of the model, based on the points that each variable achieved, the total points would be calculated according to the nomogram, and the probability of survival rate could be determined.
